# Supplementary material for: Pyrrolizidine Alkaloids in Food on the Italian Market
Source: Molecules. 2023 Jul 11;28(14):5346. doi: 10.3390/molecules28145346 (PMC10385305; doi:10.3390/molecules28145346)
Supplement: Supplementary file 1 [file molecules-28-05346-s001.zip › Supplementary material- Figure.pdf]

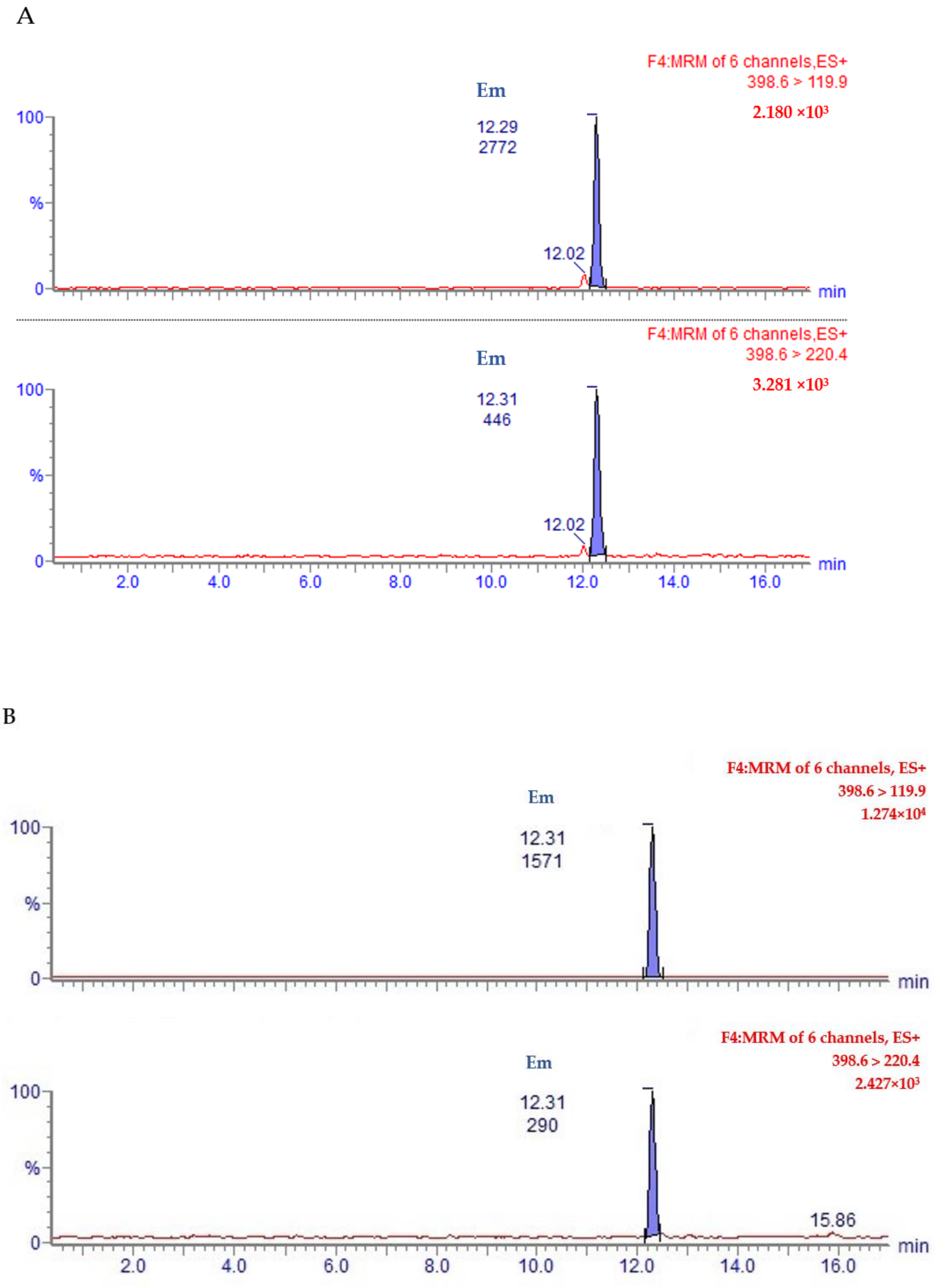

Figure S1. (A) Chromatograms of Echimidine (11 µg/kg) in a contaminated monofloral honey sample (*Stachys officinalis*); (B) Echimidine in a fortified honey sample at LOQ (1 µg/kg).

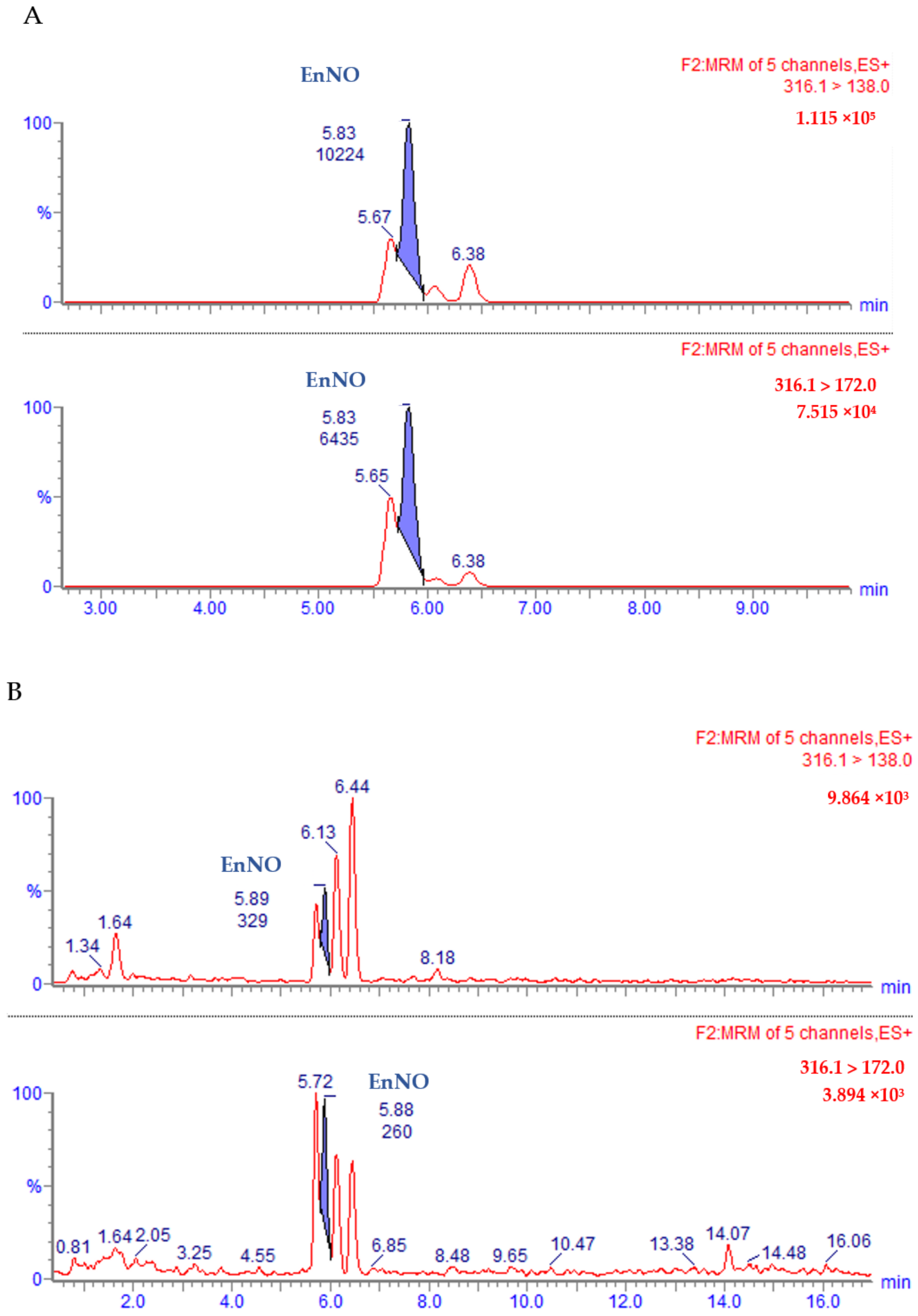

Figure S2. (A) Chromatograms of Echinatine -N-oxide (6882 µg/kg) in a positive "bee pollen" sample; (B) Echinatine-N-oxide in fortified pollen sample at LOQ (5 µg/kg).

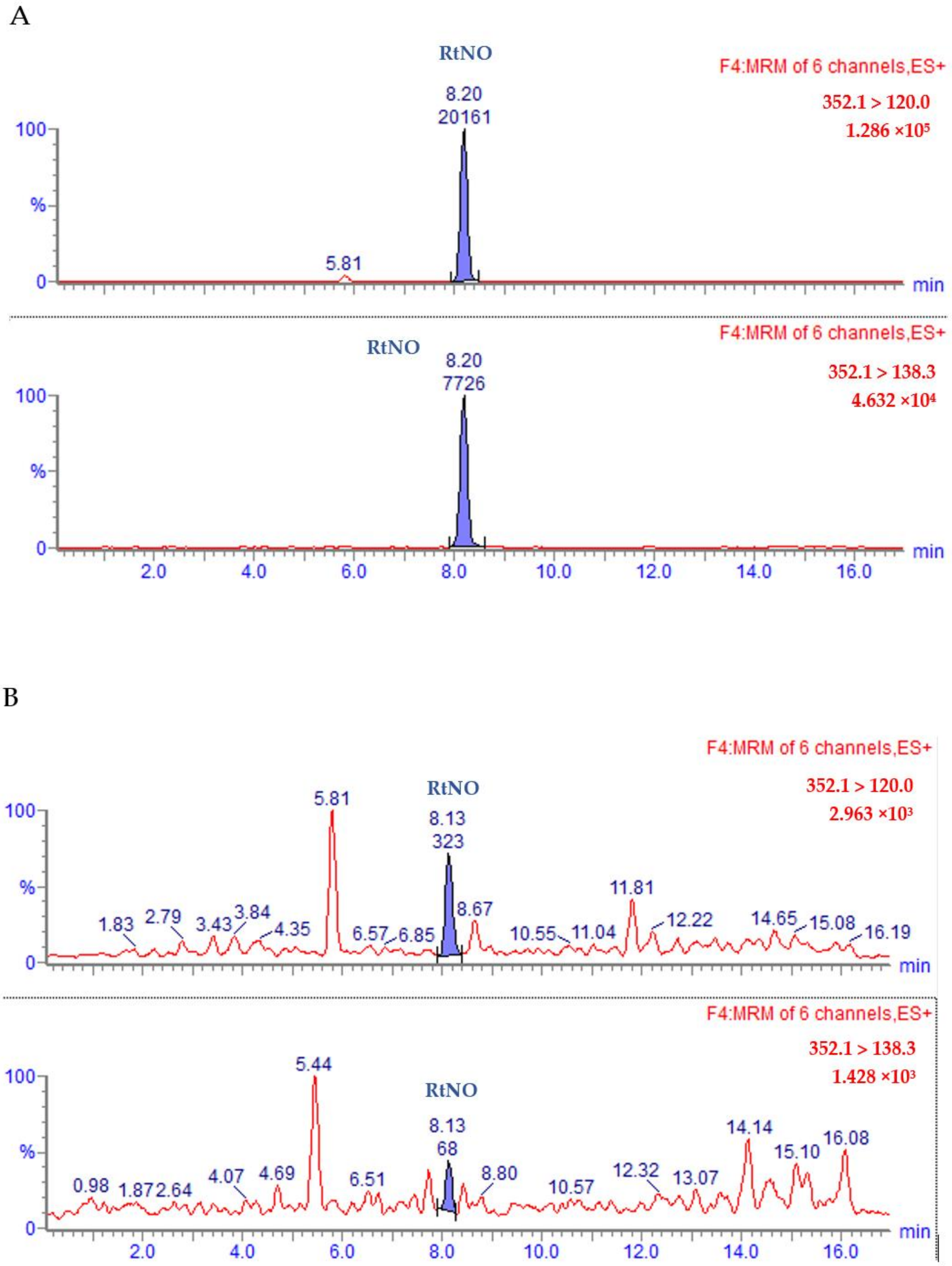

Figure S3. (A) Chromatograms of Retrorsine-N-oxide (374.2 μg/kg) in a positive "dried tea" sample (*Camellia sinensis*, green tea); (B) Retrorsine N-oxide in fortified tea sample at LOQ (5 μg/kg).

A

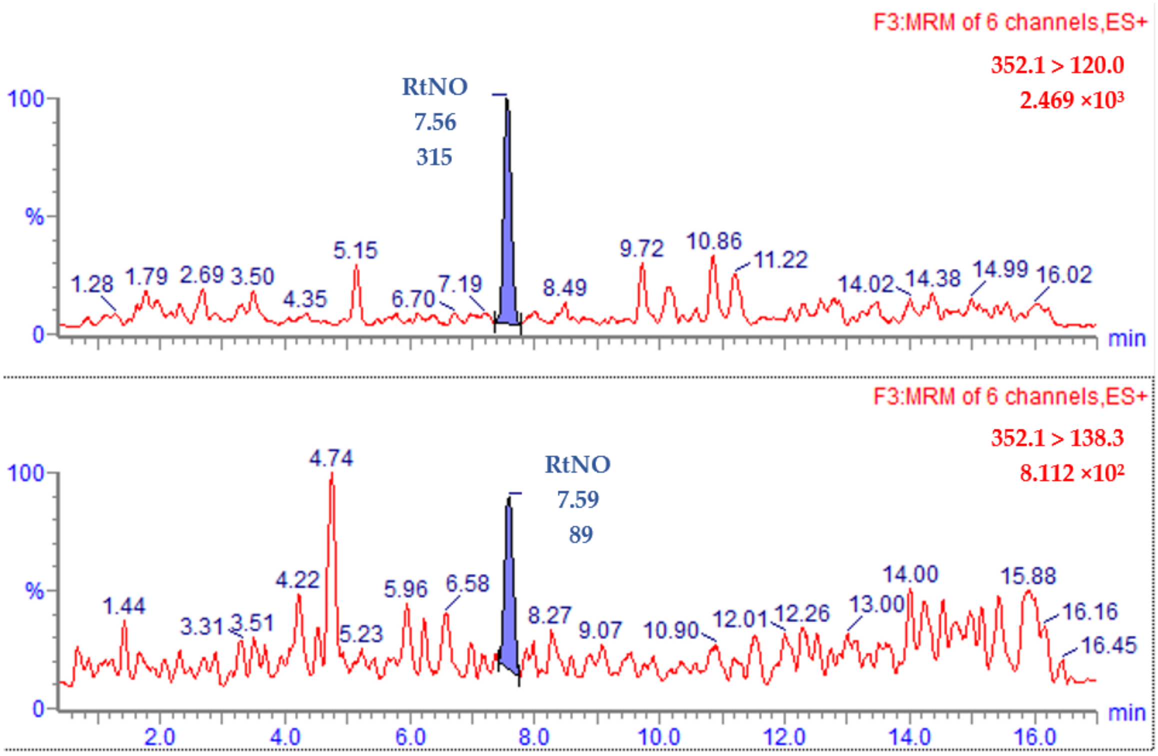

B

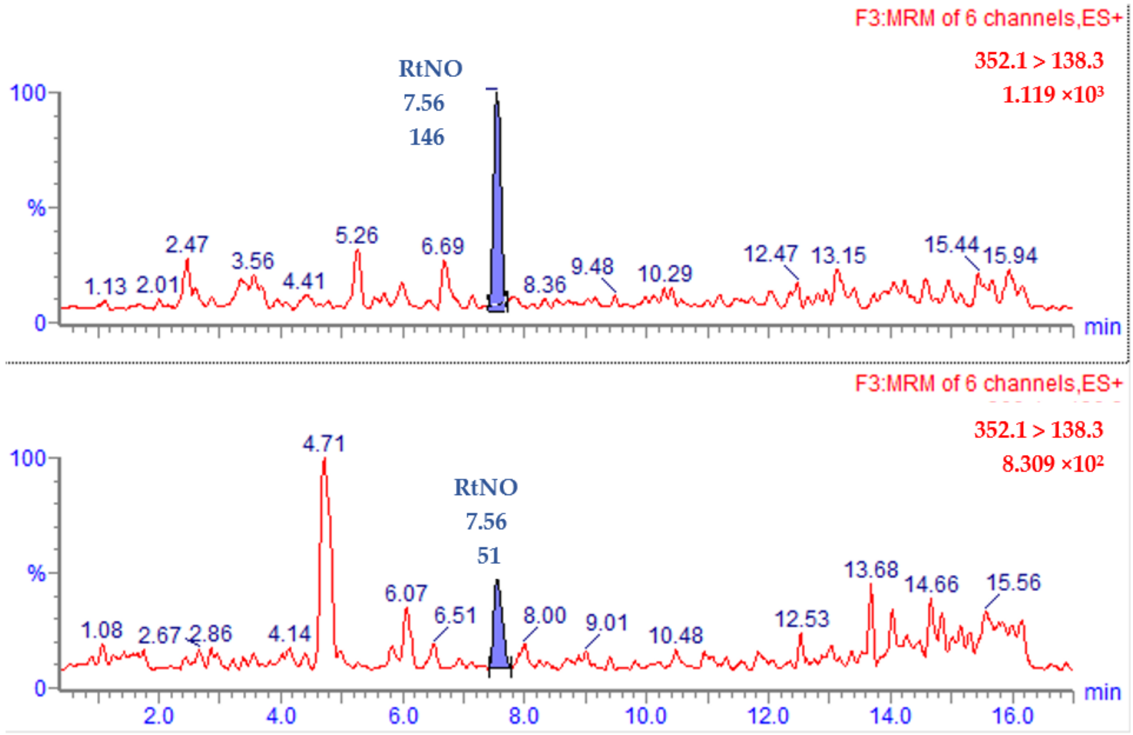

Figure S4. (A) Chromatograms of Retrorsine-N-oxide (10 µg/kg) in a positive "dried herbal infusion" sample (Chamomile); (B) Retrorsine-N-oxide in fortified dried herbal infusion sample at LOQ (5 µg/kg).

A

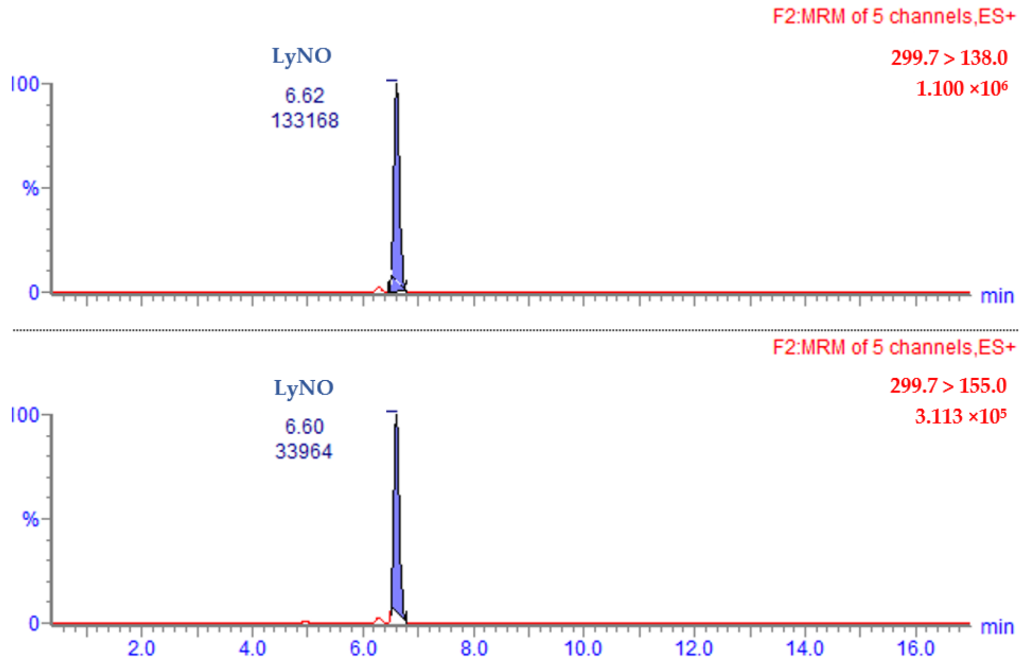

B

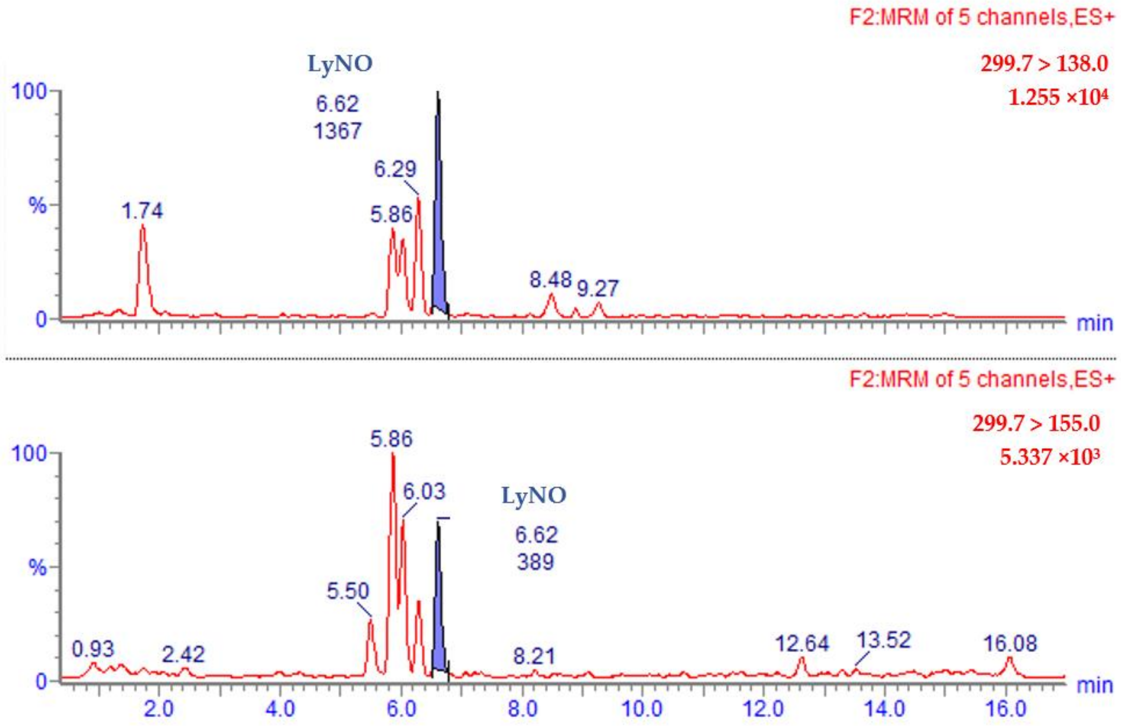

Figure S5. (A) Chromatograms of Lycopsamine-N-oxide (673.7 µg/kg) in a positive "dried herbs" sample (oregano); (B) Lycopsamine-N-oxide in fortified dried herbs sample at LOQ (5 µg/kg).

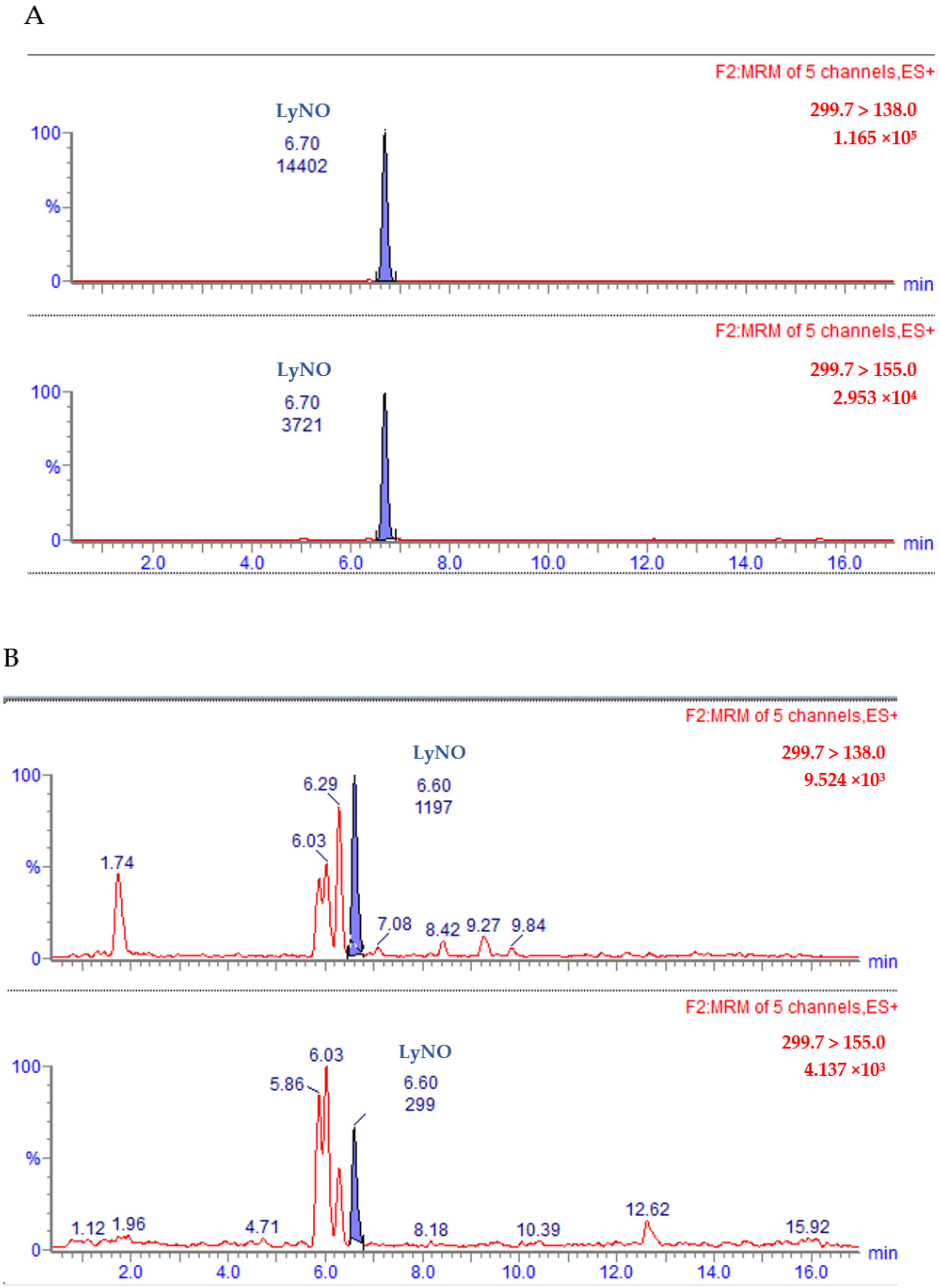

Figure S6. (A) Chromatograms of Lycopsamine-N-oxide (3410 µg/kg) in a positive "fresh borage leaves" sample; (B) Lycopsamine-N-oxide in fortified borage sample at LOQ (5 µg/kg).
