# Supplementary material for: Pyrrolizidine Alkaloids in Food on the Italian Market
Source: Molecules. 2023 Jul 11;28(14):5346. doi: 10.3390/molecules28145346 (PMC10385305; doi:10.3390/molecules28145346)
Supplement: Supplementary file 1 [file molecules-28-05346-s001.zip › Supplementary material- Table.pdf]

Table S1. Validation Parameters: mean recovery % for individual analytes and PAs/PANOs' sum.

Analytes	Mean Recovery Pollen	Mean Recovery Dried tea[1]	Mean Recovery Honey
Em-Hs	94.9%	87.9%	85.9 %
EmN	96.2%	95.0%	77.1%
Eu	101.7%	95.7%	74.2 %
EuN	87.9%	95.7%	78.3%
Ht	106.3%	95.8%	78.9 %
HtN	96.1%	99.3%	81.2%
Im	97.6%	95.3%	70.8 %
ImN	89.9%	92.7%	70.8%
Lc	103.5%	86.6%	84.7 %
LcN	87.9%	99.0%	74.6%
Ly, Id, En, Rn	96.6%	93.1%	74.4 %
LyN, IdN	94.4%	87.9%	70.2%
Rt, Us	91.1%	89.9%	70.7%
RtN, UsN	88.9%	99.4%	76.4%
Sp, St	98.8%	80.3%	82.5%
SpN, StN	89.3%	79.5%	71.7%
Sn, Ir	95.0%	87.6%	87.4%
SnN	87.2%	100.6%	79.3%
Sv	92.5%	91.9%	77.3%
SvN	98.5%	97.7%	89.4%
Sk	93.6%	96.1%	80.3%
EnN	99.2%	98.3%	76.4%
RnN	98.6%	97.6%	71.8%
IrN	87.5%	94.9%	82.4%
HsN	96.3%	93.7%	89.7%
**PAs sum**	94.8%	82.1%	77.5%

[1] this food category includes: dried herbal infusions, dried herbs, dried herbs and fresh borage leaves

Table S2. Validation results at three spiking levels for the determination of PAs/PANOs, expressed as a sum, in the three food categories.

	**Pollen**			**Dried tea[1]**			**Honey**		
**PAs/PANOs sum**	**125 µg/kg**	**12500 µg/kg**	**25000 µg/kg**	**3.75 µg/kg**	**25 µg/kg**	**125 µg/kg**	**25 µg/kg**	**250 µg/kg**	**625 µg/kg**
**sd[2]**	2.61	509.91	469.50	0.17	0.54	3.20	0.63	8.09	9.02
**Conc. mean**	118.59	11260.62	21671.61	3.22	21.77	102.95	20.83	182.83	452.31
**Repeatability[3] (RSDr-CV%)**	2.20	4.53	2.17	5.40	2.49	3.11	3.04	4.43	1.99

[1] this food category includes: dried herbal infusions, dried herbs, dried herbs and fresh borage leaves, [2]standard deviation; [3]The Coefficient of Variation (CV%) of the repeatability was calculated by analyzing the matrices in six replicates at the three fortified levels.
